# Dual conjugation of magnetic nanoparticles with antibodies and siRNA for cell-specific gene silencing in vascular cells

**DOI:** 10.3389/fddev.2024.1416737

**Published:** 2024-08-15

**Authors:** Katarzyna Karpinska, Lin Li, Tao Wang

**Affiliations:** ^1^ School of Biological Sciences, Faculty of Biology, Medicine and Health, The University of Manchester, Manchester, United Kingdom; ^2^ Department of Engineering for Sustainability, School of Engineering, The University of Manchester, Manchester, United Kingdom

**Keywords:** magnetic iron nanoparticles, siRNA, gene silencing, gene delivery, polyethyleneimine, endothelial cells, vascular smooth muscle cells, dual conjugation of nanoparticles

## Abstract

RNA therapy is a rapidly expanding field and has great promise in achieving targeted gene silencing and contributing to personalized medicine. However, the delivery of RNA molecules into targeted organs or cells is still challenging. To overcome this hurdle, a number of nanocarriers with pros and cons have been developed. This study was designed to develop a simple and cost-effective approach to functionalize biodegradable magnetic iron nanoparticles (MNPs) for cell-specific siRNA delivery. MNPs were synthesized based on co-precipitation and further functionalized with sodium citrate and polyethyleneimine (PEI) followed by material characterization using TEM, FTIR, and Zeta potential. The citrate and PEI-coated MNPs were further conjugated with CD31 antibody and complexed with siRNA using a linker-free approach. siRNA-loaded MNPs successfully knocked down the expression of *GAPDH* in human endothelial cells (ECs) and *NOTCH3* in human vascular smooth muscle cells (VSMCs). In an EC and VSMC co-culture system under shear stress to mimic blood flow, siRNA and CD31 conjugated MNPs specifically targeted and delivered siRNA into the ECs. Our approach represents a versatile platform that could be adopted for targeted general siRNA delivery.

## 1 Introduction

RNA-based therapy is a rapidly growing field and offers exciting potential for targeted and personalized drug development, contributing to precision medicine (Zhu et al., 2022; Sparmann and Vogel, 2023). RNA interference (RNAi) has emerged as a powerful and widely used method for silencing the expression of a detrimental gene in the onset or progression of a disease (Elbashir et al., 2001; Hu et al., 2020; Friedrich and Aigner, 2022). Chemically synthesized small interfering RNA (siRNA) binds upon cellular processing to its complementary messenger RNA (mRNA) sequence, causing gene-specific degradation of the target mRNA (Hu et al., 2020). By encapsulation in a nanocarrier, siRNA can be successfully protected from degradation in biological fluids, facilitating its delivery efficiency into target cells (Damase et al., 2021; Paunovska et al., 2022). Modifications of the nanocarriers by molecules, such as antibody or aptamers, that recognize target cells can achieve cell-specific drug delivery, including siRNA (Johnston and Scott, 2018; Li et al., 2023).

Small interfering RNAs are double-stranded, negatively charged molecules that typically cannot easily cross plasma membranes (Babu et al., 2016), thus requiring carriers for the efficient transport of siRNA into cells. Different materials have been designed as nonviral vectors to overcome this challenge (Paunovska et al., 2022). The selected delivery system is ideally nano in size, as factors including carrier size and surface charge have strong influence on the clearance of the carriers (Berger et al., 2024). Arami et al. (2017) report that 20–40 nm particles are better suited for siRNA delivery. A safe and effective *in vivo* siRNA delivery system is a prerequisite for siRNA-based therapeutics. Many of the current siRNA delivery technologies employ lipid, polymer, carbon, and inorganic nanoparticles (Paunovska et al., 2022) that can electrostatically interact and condense siRNA molecules to protect the siRNA from enzymatic degradation, facilitate cellular internalization, and target specific intracellular compartments. A desirable nanocarrier requires low toxicity, high loading capacity, biocompatibility, ease of preparation, and low cost (Putnam, 2006; Dave et al., 2017; Nguyen et al., 2018). Despite efforts, a perfect nanocarrier for siRNA delivery has not been identified. Limitations of current nanocarriers include biocompatibility, stability, large-scale fabrication, cost efficiency, and precision targeting. In this regard, magnetic iron oxide (Fe_3_O_4_) nanoparticles represent an alternative candidate nanocarrier.

Magnetic nanoparticles (MNPs) can be prepared in smaller sizes by simple chemical reactions (Reddy et al., 2012) and are biodegradable (Nosrati et al., 2019). The surfaces of MNPs can be modified with different types of polymer that could help covalently or non-covalently attach biomolecules and protect them from degradation. Coating MNPs with polymers, such as chitosan or polyethyleneimine (PEI), confers a net positive surface charge which helps electrostatic interaction with siRNA via NH_2_-groups (Liu et al., 2011; Wang et al., 2016). Moreover, such polymer coatings present multiple binding sites for ligands. A successful example is the *NOTCH1* siRNA-loaded and PEI-coated MNPs which have been preferentially taken into breast cancer cells with high efficiency and which have inhibited cell proliferation and increased apoptosis and cell death (Yang et al., 2014). Thus, iron oxide MNPs can be exploited as an efficient non-viral delivery system for gene therapy.

Cell-type specific targeting is desirable for the delivery of therapeutic molecules into diseased cells in a tissue; however, this functionality is currently limited for the PEI-functionalized MNPs. Antibody-conjugated nanoparticles offer a promising platform for tissue or cell-type-specific drug delivery, but studies regarding the modification of MNP with antibodies and siRNA simultaneously for specific targeting are lacking. Additionally, iron oxide MNPs have super paramagnetism (SPIO) (Li et al., 2017). This property aids in the targeted and image-guided delivery (magnetofection) of therapeutic siRNA when an external magnetic field is applied to achieve site-specific enrichment at a desired region of the tissue, thereby reducing the accumulation and toxicity of siRNA in healthy tissues (Scherer et al., 2002; Namiki et al., 2009). Thus, the SPIO property of MNPs confers additional benefit for the targeted delivery of siRNA.

Cardiovascular diseases are the leading worldwide cause of morbidity and mortality. There is a pressing need to develop precise and targeted treatment to deliver therapeutic molecules into specific vascular cell types based on disease pathologies. Thus, in this study we developed a novel and simplified dual conjugation method to functionalize Fe_3_O_4_ MNPs with citrate and PEI, which then link with specific antibodies and siRNA for cell-specific delivery of siRNA.

## 2 Materials and methods

### 2.1 Synthesis of iron oxide nanoparticles

Magnetic nanoparticles were prepared using a modified chemical co-precipitation method by co-precipitating ferrous and ferric ion salts in an alkaline solution. Iron(II) chloride tetrahydrate (FeCl_2_·4H_2_O) and iron(III) chloride hexahydrate (FeCl_3_·6H_2_O) (Thermo Fisher Scientific, United Kingdom) were used. We mixed 0.78 g FeCl_3_·6H_2_O and 0.44 g FeCl_2_·4H_2_O in a 2:1 M ratio and then dissolved it in 50 mL distilled water. The pH of the reaction was adjusted to 10 using 30% ammonia hydroxide solution. The sample was heated at 80 °C for 4 h while stirring and then cooled to room temperature. The reaction is represented by the following formula:

$$2{\text{Fe}}^{3+}+{\text{Fe}}^{2+}+8{\text{OH}}^{-}\;\to \;{\text{Fe}}_{3}O_{4}↓+4H_{2}O$$

The black Fe_3_O_4_ NPs formed in the reaction were magnetically separated, washed with distilled water and ethanol three times, and then dried in a vacuum at 40 °C.

### 2.2 Functionalization with trisodium citrate

To add carboxylic groups to MNPs, 20 mg of the above prepared dried MNPs were dispersed in 10 mL of dH_2_O, and the solution was added dropwise into 50 mL of 0.02 M trisodium citrate dihydrate (C₆H₅Na₃O₇·2H₂O) (Thermo Fisher Scientific, United Kingdom) solution followed by stirring for 2 h at 80 °C. Citrate-modified nanoparticles (NMP@Cit) were collected by external magnet, washed thrice with dH_2_O to remove free sodium citrate, and used for material characterization.

### 2.3 Functionalization of MNP@Cit with polyethyleneimine (PEI)

Citrate-modified NPs were further functionalized with cationic polymer polyethyleneimine (PEI) (branched, MW 25,0000) purchased from Fisher Scientific (United Kingdom). Totals of 0.5, 1.0, and 1.5 mL of PEI solution (150 mg/mL) were added dropwise under mild sonication into separate containers with 2 mL of MNP@Cit (2 mg/mL). This resulted in three different citrate–PEI mass-ratios: Formulation-0.5 (1:19), Formulation-1.0 (1:38), and Formulation-1.5 (1:57). After further sonication for 5 min and mixing 500 rpm for 2 h at room temperature (RT), the nanoparticles were collected by centrifugation at 14,800 rpm for 45 min and washed three times with nuclease-free water. The resulting MNP@Cit@PEI nanoparticles were then resuspended into the desired concentration and stored at 4°C.

### 2.4 MNP@Cit@PEI conjugation with antibody and complex with siRNA

We mixed 1 mL of 40 μg/mL of MNP@Cit@PEI in nuclease free water with 0.83 µL of 0.5 mg/mL Anti-CD31 antibody Alexa Fluor^®^ 647 (Abcam, United Kingdom) and stirred for 10 min at RT to form MNP@Cit@PEI@Ab-A647. After this, 50 pmoL of Silencer™ Cy™3-labeled GAPDH siRNA against human/mouse/rat sequences (Thermo Fisher Scientific, United Kingdom) were added to the samples and incubated 20 min to form MNP@Cit@PEI@Ab-A647@siRNA-Cy3. The nanoparticles were collected using centrifugation at 14.800 rpm for 20 min. The samples were then washed thrice in nuclease-free water and resuspended in serum-free cell culture media for the cell studies below.

### 2.5 Instrument characterization

Transmission electron microscopy (TEM) was used to determine the size and shape of the MNPs. TEM images were obtained using FEI Tecnai 12 BioTwin TEM operated at 200 kV. A total of 10 μL of the samples was placed on top of a carbon-coated copper grid of 200 meshes. The samples were then evaporated naturally before examination.

Fourier-transform infrared spectroscopy (FTIR) measurements were performed at room temperature on a Nicolet Nexus 670 FT-IR spectrometer (United Kingdom). All spectra were taken via the attenuated total reflection method with a resolution of 4 cm−1 and 60 scans. Samples were dried in vacuum at 40 °C before the analysis.

Zeta potential measurements were performed to confirm surface fictionalization of the nanoparticles produced. Zeta (ζ) potential was measured by light scattering via a Zetasizer Nano ZS (Malvern Instruments, United Kingdom). The experiments were performed with aqueous solutions of NPs, pH 7.0. The final concentration of the nanoparticles was 200 μg/mL. All data were recorded with at least six runs.

The vibrating sample magnetometer (VSM) SQUID system (Quantum Design MPMS 3) was used to measure the magnetic properties of the MNPs operating at 300 K at room temperature.

### 2.6 Cell culture

Human coronary artery smooth muscle cells (hCASMCs) were cultured in smooth muscle growth media 2, supplemented with fetal calf serum, insulin, epidermal growth factor, and basic fibroblastic growth factor (PromoCell, Germany) (Kelleher et al., 2019). Human coronary artery endothelial cells (hCAECs) were cultured in endothelial cell growth media supplemented with fetal calf serum, epidermal growth factor, basic fibroblast growth factor, insulin-like growth factor, vascular endothelial growth factor, ascorbic acid, heparin, and hydrocortisone (PromoCell, Germany) (Kelleher et al., 2019). Both sub-cultured primary cell lines (hCASMC and HCAEC) were used before the tenth passage for downstream applications. All cells were grown on 37 °C and 5% CO_2_ and passaged when cell confluency reached 80%–90%. HCASMCs were seeded into 24-well plate at a density of 80,000 cells/mL and monitored until cells reached ∼80% confluency. HCAECs were seeded into 24-well plates at a density of 80,000 cells/mL and monitored until cells reached ∼80% confluency.

### 2.7 Agarose gel electrophoresis of RNA

Complex formation between MNP@Cit@PEI and siRNA was confirmed by gel electrophoresis using 2% agarose gel. Nanoparticle complexes (with weights of 4, 7, 10, 20, 40, and 60 µg) were prepared with 20 µL 50 pmoL of siRNA in RNase-free water at RT for 30 min. Naked siRNA was used as a control. The samples were loaded into the gel, and electrophoresis was carried out at 100 V for 10 min in TAE buffer (Tris base, acetic acid, and EDTA, pH 8.3). The gel was stained with SYBR GREEN II RNA gel stain (Thermofisher, United Kingdom) for 25 min at RT. Safe view was used to visualize siRNA bands using a UV transilluminator at 365 nm.

### 2.8 PrestoBlue™ cell viability assay

PrestoBlue™ cell viability reagent is a ready-to-use reagent for rapidly evaluating the viability and proliferation of a wide range of cell types. A PrestoBlue assay was performed according to the manufacturer’s protocol (Thermo Fisher Scientific, United Kingdom). Briefly, cells were seeded at 10,000 cells/well in flat-bottomed, 96-well plates 24 h before treatment. Once confluent, cells were washed with 1x PBS; 100 µL of serum free media containing MNPs with final concentrations of 20, 40, 60 and 80 μg/mL were then added into the wells in triplicate. To correct the background, the control wells containing only culture medium with MNPs were included. Non-treated cells were used as a positive control. The cells were incubated for 4 h in a CO_2_ incubator at 37 °C. Thereafter, the medium was changed for serum-containing media and the cells were further incubated for 24 and 48 h. After specific time, the cells were washed with 1x PBS, and then 10 µL of Presto Blue™ reagent was added to each well and incubated for 10 min at 37 °C. Fluorescence reflecting cell viability was read at excitation 530 nm and emission 590 nm using Microplate Reader Biotek Synergy HT. Cell viability was expressed as a percentage relative to the non-treated cells.

### 2.9 Cellular uptake of MNPs–fluorescent microscopy

HCAEC cells (200,000/well) were cultured in a 24-well plate for 24 h until confluent. They were washed with 1x PBS and then incubated with MNP@Cit@PEI@Ab-A647@siRNA-Cy3 complexes (40 μg/mL) for 4 h and 24 h. SiRNA-Cy3 alone was used as a positive control. Thereafter, the cells were washed three times with PBS and then fixed by 4% paraformaldehyde. The nuclei of the cells were stained by 4′,6-diamidino-2-phenylindole (DAPI). Images were taken with a fluorescence microscope Olympus using ×63 oil immersion objective.

### 2.10 Cellular uptake of MNPs–ImageStream flow cytometry

To quantify the cellular uptake of the siRNA and antibody conjugated MNPs, hCAECs (200,000/well) were cultured in 24-well plates for 24 h, and then incubated with the MNPs complexes (40 μg/mL) for 2.5 h. After a thorough wash with PBS, cell pellets were treated with trypsin-EDTA and collected by centrifugation at 500 g. We added 50 µL of 4% paraformaldehyde to the cell pellet followed by 10 min incubation at room temperature. The cells were washed twice with PBS and centrifuged at 800 *g* for 5 min to collect all cells. The samples were analyzed using ImageStream®X Mark II Imaging Flow Cytometer. The acquisition of >5,000 events was collected using a 40 mW Red laser (640 nm) and 670/14 bandpass filter.

### 2.11 Co-culture hCAECs and hCASMCs in the ibidi pump system

HCASMCs (100,000 cells in 100 µL) were first seeded onto the ibidi µ-Slide I Luer slides and incubated in CO_2_ incubator at 37 °C for 24 h until confluent. The cells were washed with PBS, and then 1 mg/mL ice cold Matrigel was pipetted onto the hCASMCs and incubated for 1 h at 37 °C before the solution was aspirated. HCAECs suspensions (200,000 cells in 100 µL) were then seeded onto the Matrigel-coated hCASMCs and cultured in a CO_2_ incubator at 37°C for 24 h. The µ-slide was then connected to the ibidi pump system with a shear stress set at 10 dyn/cm^2^ and cultured for 24 h.

### 2.12 Immunocytochemistry

Cells were rinsed with PBS and fixed with 4% paraformaldehyde (PFA) for 10 min. They were then washed thrice with PBS and incubated with 10% donkey serum in PBS to block non-specific binding for 1 h prior to incubation with primary antibodies for 1 h at room temperature. Primary antibodies VE-cadherin mouse IgG and calponin rabbit IgG (Abcam, Cambridge, United Kingdom) were diluted 1:200 in 10% donkey serum. Cells were then washed three times with PBS and further incubated with secondary antibody Alexa Fluor^®^ 488 Donkey Anti-Mouse and Alexa Fluor^®^ Cy3 Donkey Anti-Rabbit (Abcam, Cambridge, United Kingdom 1:500 dilution in 10% donkey serum) for 1 h at room temperature. After additional washing with PBS, cell nuclei were stained with 1 μg/mL DAPI for 5 min. Samples were left to dry at room temperature and slides mounted with ProLong gold antifade reagent (Thermo Fisher Scientific, United Kingdom).

### 2.13 *In vitro* transfection

HCAECs (200,000 cells/well) were grown in 24-well plates for 24 h and then incubated with either siRNA (100 pmoL/well) or MNP@Cit@PEI@siRNA in 500 μL of serum-free media for 4 and 24 h, respectively. Thereafter, the media were replaced with a 500 μL medium containing 20% fetal bovine serum, and cells were incubated at 37 °C in 5% CO_2_ incubator for an additional 48 h. Thence, cells were washed and collected for downstream analysis.

### 2.14 RT-qPCR

Total RNA was extracted from cells using RNeasy Mini Kit (Qiagen, United Kingdom). A NanoDrop (NanoDrop spectrophotometer ND-1000; Thermo Scientific, United Kingdom) was used to determine RNA concentrations. The RNA samples were reverse transcribed into cDNA using a High-Capacity RNA-to-cDNA™ Kit (Applied Biosystems, United Kingdom) according to the manufacturer’s protocol. After the cDNA was synthesized, PCR was performed with Power SYBR™ Green PCR Master Mix (Applied Biosystems, United Kingdom) using a CFX96 Touch Real-Time PCR System (BioRad, United Kingdom). The sequences of the primers used for the human *GAPDH* gene were 5′-GAA​ATC​CCA​TCA​CCA​TCT​TCC​AG-3′ and 5′-GAG​CCC​CAG​CCT​TCT​CCA​TG-3′. Human 18S rRNA, a housekeeping gene, was used as an internal control to normalize the *GAPDH* gene expression. Human 18S rRNA mRNA was amplified using 5′-GGC​CCT​GTA​ATT​GGA​ATG​AGT​C-3′ and 5′-CCA​AGA​TCC​AAC​TAC​GAG​CTT-3’ primers (Eurofins, United Kingdom). The primers used for *NOTCH3* qPCR were 5′ CAT​CTC​CGA​CCT​GAT​CTG​CC 3′ and 5′ GTC​TGT​AGA​GCG​GTT​TCG​GA 3’.

### 2.15 Statistics

All experiments were performed at least thrice. Data were presented as mean ± standard error (SEM). Comparisons of the two groups were made using an unpaired *t*-test. For the comparison of datasets above two samples, one-way or two-way ANOVA with Tukey’s multiple comparisons *post hoc* tests were used.

## 3 Results

### 3.1 Generation and characterization of MNPs

**FIGURE 1:**
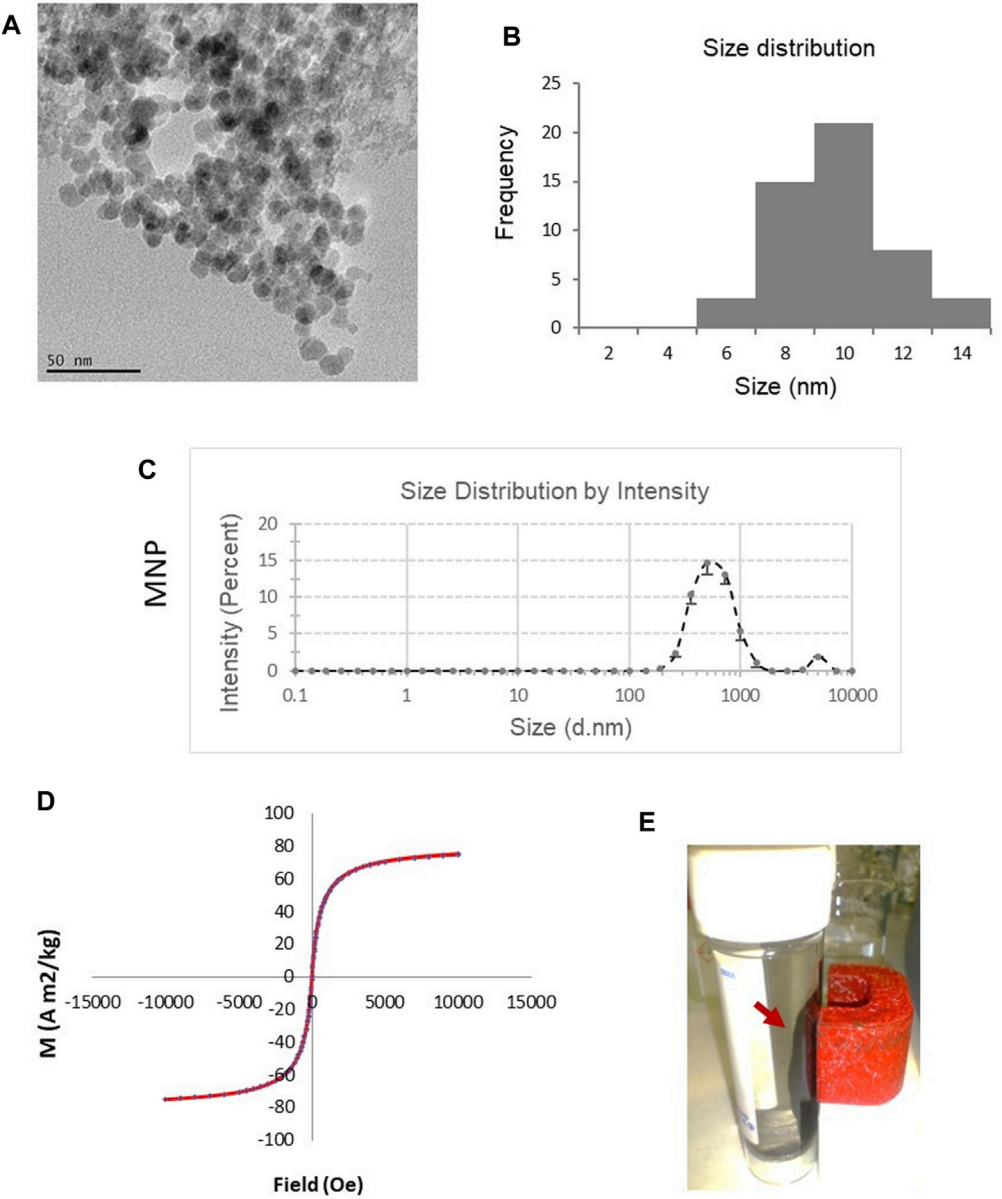
Characterization of magnetic Fe_3_O_4_ NPs (MNPs) prepared by chemical co-precipitation. **(A)** TEM image of the MNPs. **(B)** Size distribution of the cores of the MNPs based on TEM. **(C)** Hydrodynamic size of MNPs measured by dynamic light scattering (DLS). Data are mean ± SE from three readings. **(D)** Magnetic curve of MNPs determined using vibrating sample magnetometer SQUID system. **(E)** MNPs in water were collected (red arrow) by a magnet, demonstrating theirmagnetic properties.

Fe_3_O_4_ MNPs were prepared by co-precipitating ferrous and ferric ion salts in an alkaline solution at room temperature. TEM characterization of the MNPs showed a roughly spherical morphology (Figure 1A). The core size of the MNPs measured by TEM imaging ranged from 6 to 14 nm, with an average of 10 nm (Figure 1B). The major peak of the hydrodynamic size measured by dynamic light scattering (DLS) was 538 ± 56 nm (Figure 1C). The magnetic properties of the MNPs were evaluated using VSM-SQUID. The magnetization curve indicated superparamagnetic behavior with an absence of remnant effect and the saturation magnetization to be 80.1 A m2/kg (Figure 1D). The magnetic property was also directly observed by the collection of the MNPs in water when a magnet was applied (Figure 1E). When the external magnetic field was removed, the magnetic nanoparticles could be well dispersed by gentle shaking, which is a critical property of MNPs in biomedical and bioengineering applications.

### 3.2 Functional modification of MNPs with citrate and PEI

**FIGURE 2:**
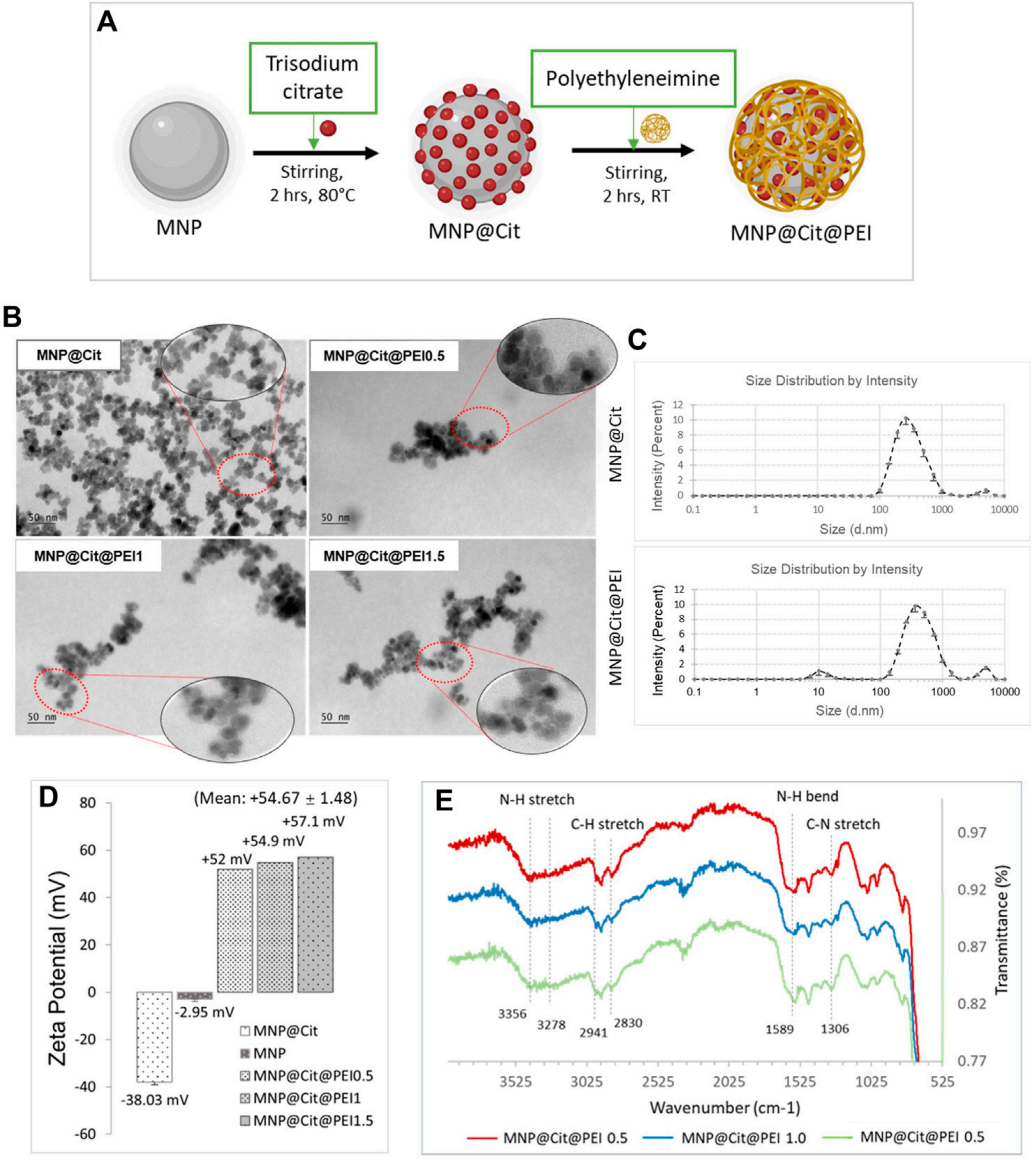
Modification and characterization of citrate- and polyethyleneimine-functionalized magnetic Fe_3_O_4_ NPs (MNPs). **(A)** Schematic diagram of MNP functionalization by trisodium citrate dihydrate and polyethyleneimine (PEI) to produce MNP@Cit@PEI. **(B)** TEM images of MNPs treated with citrate (MNP@Cit) and their further fictionalization with a different amount of PEI: Formulation 0.5, 1.0, and 1.5 (MNP@Cit@PEI0.5, MNP@Cit@PEI1.0, and MNP@Cit@PEI1.5). **(C)** Hydrodynamic size of MNP@Cit and MNP@Cit@PEI measured by dynamic light scattering (DLS). Data are mean ± SE from three readings. **(D)** Zeta potential measurements of surface charges the MNP@Cit@PEI. Data for MNP@Cit and MNP presented as mean ± SE. **(E)** TIFR analysis of MNP@Cit@PEI.

To improve the stability of the MNPs in the solution and provide a functional surface to conjugate biomolecules such as antibodies, sodium citrate modification was performed to produce MNP@Cit (Figure 2A) where negatively charged carboxyl groups were added to the MNP surface. To prepare the MNPs for complexing with siRNA, PEI was used to further functionalize the MNP surface to produce MNP@Cit@PEI (Figure 2A). PEI is a cationic polymer with a repeating unit composed of the amine group and two-carbon aliphatic CH_2_CH_2_ spacer which confers a net positive surface charge and helps electrostatic interaction with siRNA via NH_2_-groups (Boussif et al., 1995).

TEM images showed that the PEI functionalization added a thin layer around the MNPs, suggesting the fabrication of a core (iron oxide)-shell (polymer) structure (Figure 2B). When an increasing amount of PEI was used for the production of MNP@Cit@PEI, the polymer layer remained similar at ∼5 nm thick (Figure 2B). The major peaks of the hydrodynamic size of the MNP@Cit and MNP@Cit@PEI are 315 ± 19 nm and 472 ± 6 nm, respectively (Figure 2C).

Citrate coating of MNPs resulted in a significant shift of zeta potential from −2.95 mV to a more negative value of −38.03 mV. Further modification by PEI using Formulations-0.5, 1.0, and 1.5 reversed the zeta potential to high positive values of +52 mV, +54.9 mV, +57.1 mV, respectively, with an average of +54.67 ± 1.48 (Figure 2D). The higher negative and positive zeta potential values away from zero are indictive of the good colloidal stability of the MNPs.

The MNP@Cit@PEI was then characterized using FTIR spectroscopy in a wavenumber range of 4,000–400 cm^−1^ (Figure 2E). The characteristic peaks of PEI N-H stretch at 3,356 and 3,287 cm^−1^ (Nandiyanto et al., 2019; Bali et al., 2021), NH bend at 1,589 cm^−1^ (Choi et al., 2018; Bali et al., 2021), C-H stretch at 2,941 and 2,830 cm^−1^ (Choi et al., 2018; Bali et al., 2021), and C-N stretch at 1,306 cm^−1^ (Chen et al., 2006) indicated successful MNP modification by PEI.

### 3.3 *In vitro* cytotoxicity evaluation

**FIGURE 3:**
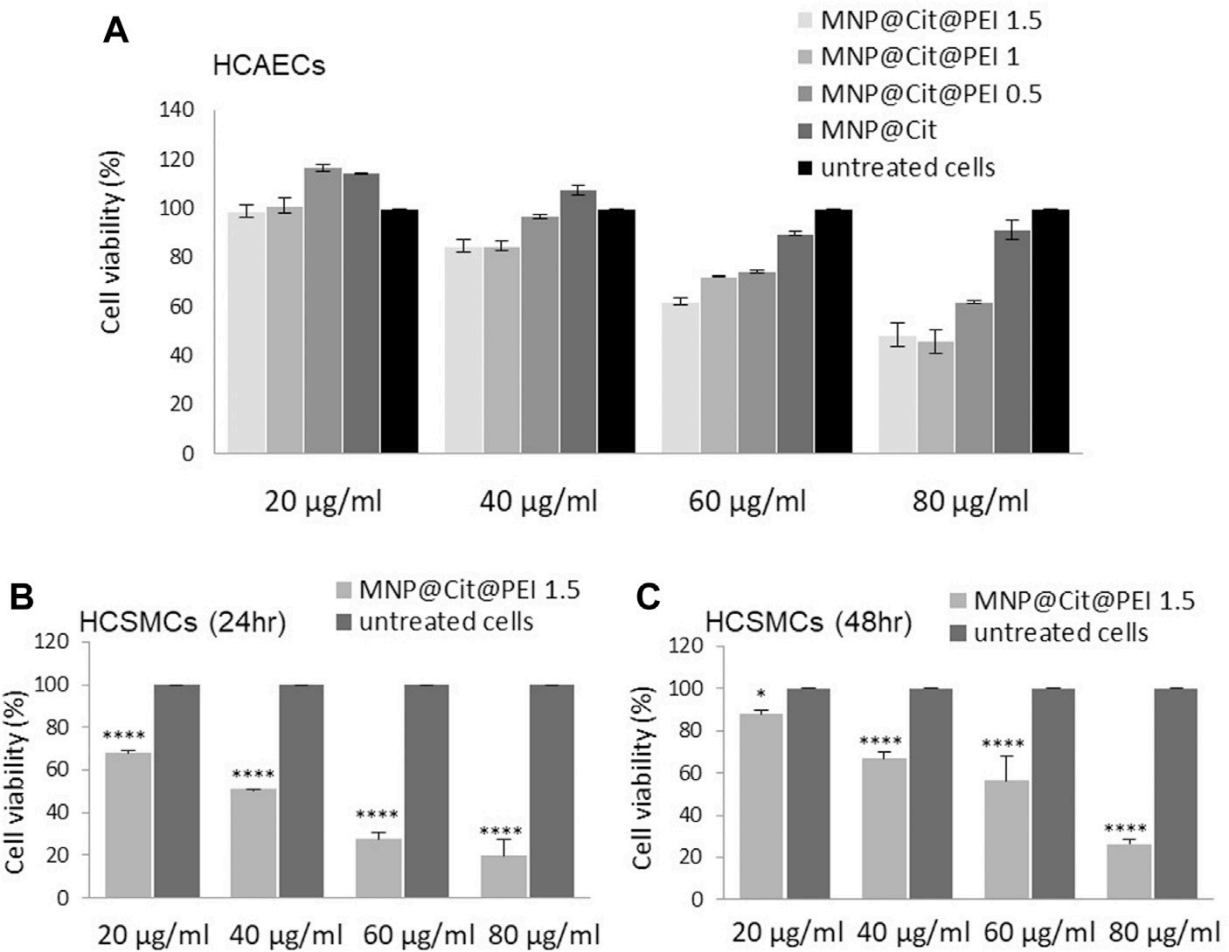
Cytotoxicity assay. Citrate- and PEI-modified magnetic Fe_3_O_4_ NPs (MNP@Cit@PEI) were incubated with HCAECs **(A)** and HCSMCs **(B, C)**, and cell viabilities were analyzed using PrestoBlue™ assay. **(A)** HCAECs were incubated with 20–80 μg/mL of MNP@Cit modified with different amounts of PEI: MNP@Cit@PEI1.5, MNP@Cit@PEI1.0, MNP@Cit@PEI0.5, and MNP@Cit for 24 h. **(B, C)** HCSMCs were incubated with 20–80 μg/mL of MNP@Cit@PEI1.5 for 24 h **(B)** and 48 h **(C)**. Cell viabilities presented as percentage of control. Data are mean ± SE, n = 3. Two-way ANOVA and Tukey’s *post hoc* test, *p < 0.05, ****p < 0.0001. PEI1.5, PEI1.0, and PEI0.5 represent 1.5, 1.0, and 0.5 mL of PEI solution (150 mg/mL) combined with 2 mL of MNP@Cit (2 mg/mL) in the MNP@Cit@PEI production process.

Citrate- and PEI-functionalized MNP@Cit@PEI were then subjected to cytotoxicity analysis before being used for the downstream conjugation of biomolecules. Human coronary artery endothelial cells (hCAECs) were cultured with different concentrations of MNP@Cit or MNP@Cit@PEI of 20–80 μg/mL, followed by PrestoBlue™ assay (Figure 3A). After 24 h of incubation with hCAECs, the MNPs at 20 μg/mL showed no toxicity for all types of nanoparticles tested (Figure 3A). Moreover, MNP@Cit@PEI produced by Formulation-0.5 at a concentration 20 μg/mL seemed to stimulate the growth of hCAECs. At a concentration of 40 μg/mL, MNP@Cit@PEI produced by Formulation-1.5 and 1.0 only slightly reduced the viability of hCAECs to around 84% (Figure 3A). A higher concentration (60 μg/mL) in Formulation-1.5 and 1.0 reduced hCAECs viability to 62% and 73%, respectively. Thus, we chose the concentration of 40 μg/mL of MNP@Cit@PEI Formulation-1.5 for the downstream experiments.

The toxicity of MNP@Cit@PEI was also determined on human primary coronary artery smooth muscle cells (hCASMCs), which showed MNP dose-dependent cellular toxicity. (Figures 3B, C). After 24-h treatment with MNP@Cit@PEI Formulation-1.5, cell viability reduced to 68.8% ± 0.7% with 20 μg/mL MNP treatment and 51.0% ± 0.1% with 40 μg/mL MNP treatment and further reductions with higher concentrations of the MNPs used (Figure 3B). However, after prolonged 48-h treatment, cell viabilities recovered to 88.0% ± 1.8% and 66.7% ± 3.6% for the two lower concentrations of MNPs (Figure 3C), suggesting good adaptive capability of the hCASMCs to the MNP@Cit@PEI exposure.

### 3.4 Conjugation of antibodies and siRNA onto MNPs and cellular uptake of the siRNA

**FIGURE 4:**
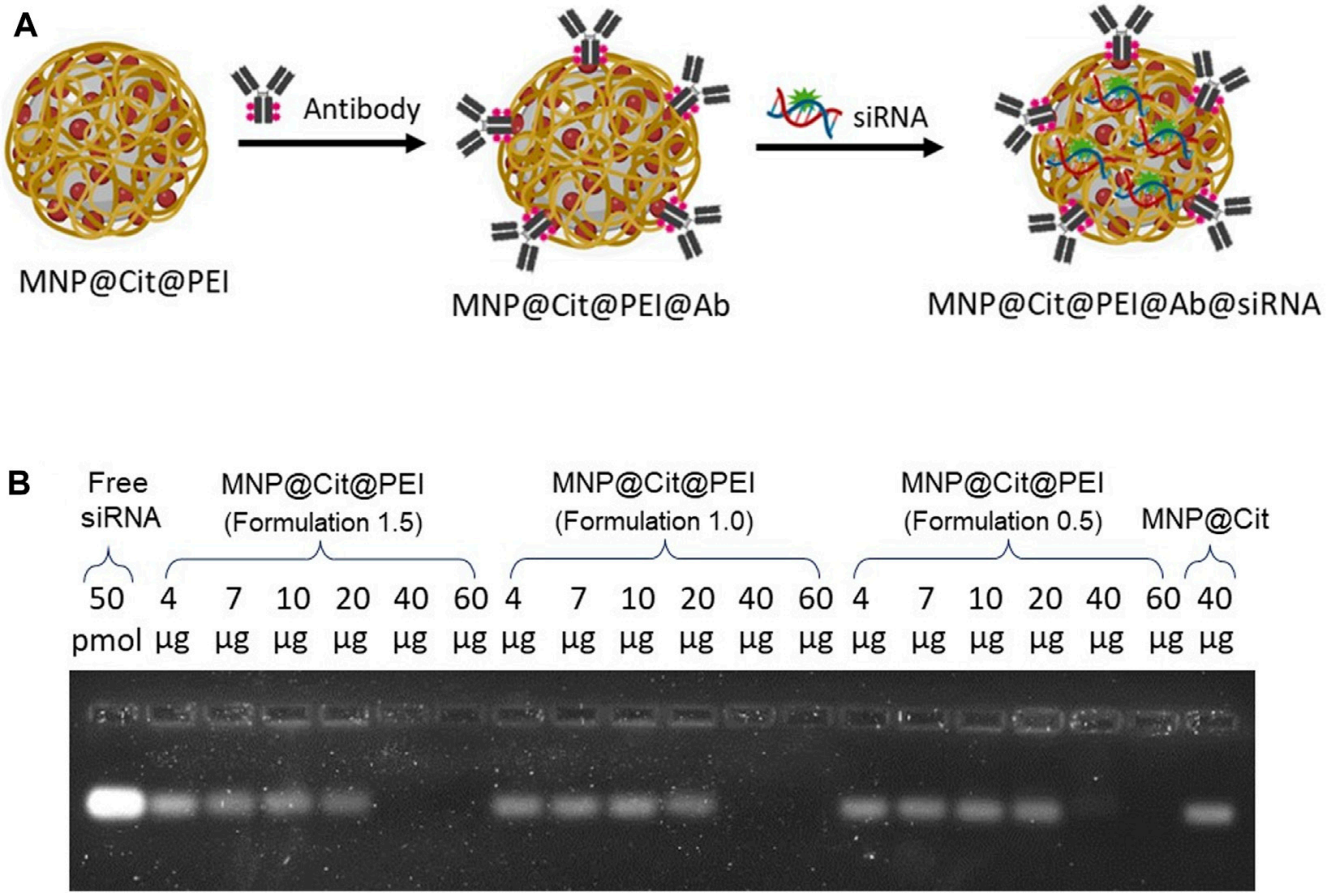
Determination of siRNA binding to citrate- and PEI-modified magnetic Fe_3_O_4_ NPs (MNP@Cit@PEI). **(A)** Schematic diagram showing overall functionalizing of MNP@Cit@PEI by antibodies and siRNA. **(B)** Different amounts (4, 7, 10, 20, 40, 60 µg) of MNP@Cit@PEI with different PEI (formulation 1.5, 1.0, and 0.5) conjugated with 20 µL 50 pmoL GAPDH siRNA. Samples were then subjected to 2% agarose gel electrophoresis and stained by SYBR GREEN II RNA gel stain to visualize unbound free siRNA. The intensity of the RNA bands reversely correlate with the conjugation efficiency. Lack of a band indicates an effective binding between siRNA and MNP@Cit@PEI.

We uploaded antibodies and siRNA onto the citrate- and PEI-functionalized MNP@Cit@PEI by a linker-free approach (Figure 4A). We first determined the efficiency of siRNA loading on PEI-modified MNPs. Agarose gel electrophoresis of the conjugation reaction was used to visualize the unbound free siRNAs that were able to migrate into the gel, which inversely reflected the binding efficiency. We incubated 50 pmoL of *GAPDH* siRNA with different amounts of MNP@Cit@PEI, ranging 4–60 µg. Results showed that stable siRNA-MNP complexes started to form when 20 µg MNPs were used in the reaction, and a nearly 100% of siRNA binding efficiency was observed (i.e., a lack of a free siRNA bend on the gel) when 40 and 60 µg MNP@Cit@PEI were used (Figure 4B).

**FIGURE 5:**
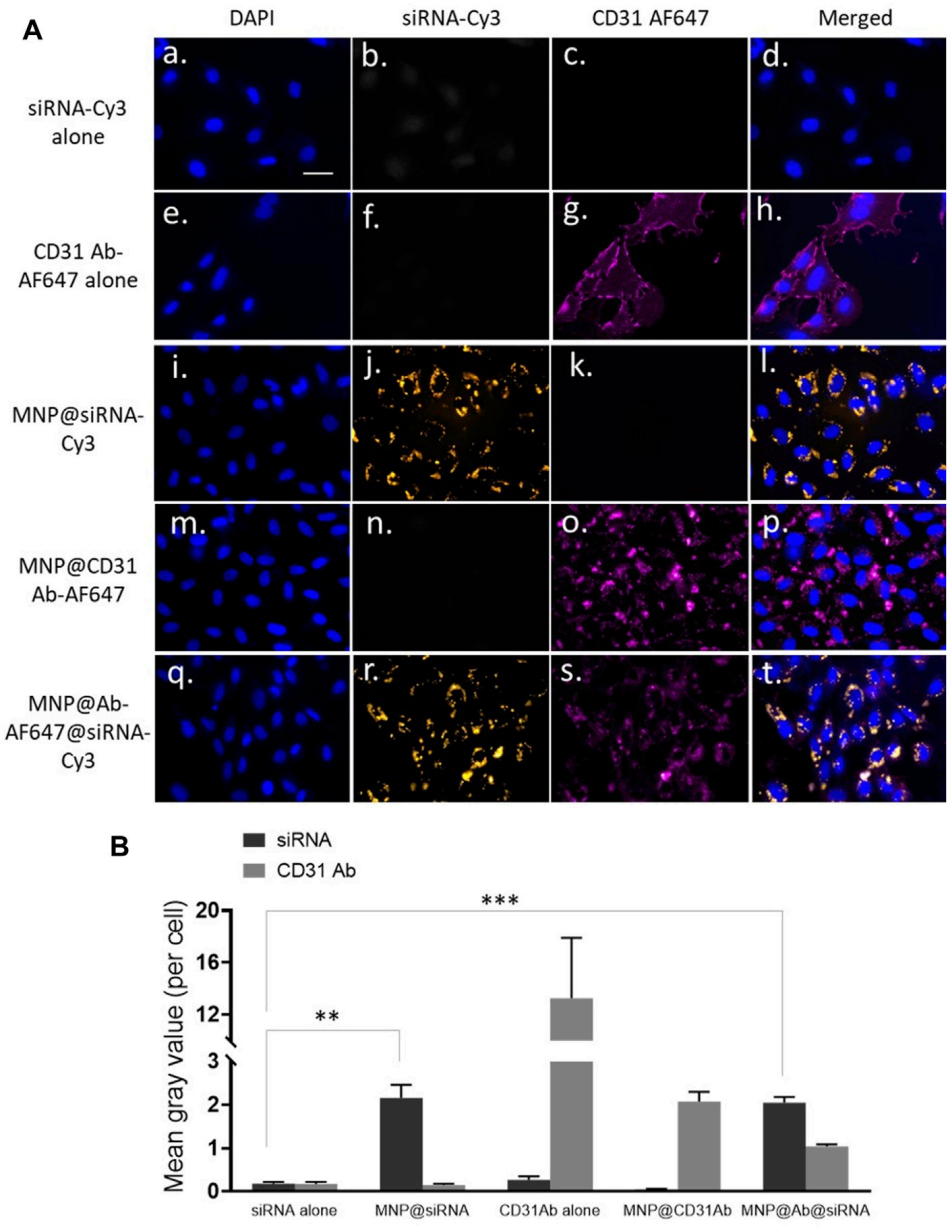
Delivery of siRNA by antibody functionalized magnetic Fe_3_O_4_ NPs. CD31 antibody-AlexaFluor647 (CD31Ab-AF647) and GAPDH siRNA-Cy3 (siRNA-Cy3) dual-loaded MNP@Cit@PEI nanoparticles (NPs@Ab-AF647@siRNA-Cy3) were incubated with hCAECs for 4 h. **(A)** Delivery of siRNA into hCAECs was visualized using fluorescence microscopy. Cell nuclei were counterstained with DAPI (blue). SiRNA-Cy3 showed yellow, and CD31 showed red. Scale bar represents 25 µm that applies to all images. **(B)** Quantification of fluorescent signals from three random views of different microscopy images, presented as mean grey value per cell. Data are mean ± SE. Two-way ANOVA and Tukey’s *post hoc* test, **p < 0.01, ***<0.001.

We aimed to deliver siRNA into endothelial cells; therefore, Alexa Fluor 647-labelled antibody specifically against endothelial marker CD31 and Cy3-labelled *GAPDH* siRNA were dual conjugated onto MNP@Cit@PEI: MNP@Cit@PEI@CD31-AF647@siRNA-Cy3. These antibody and siRNA complexed MNPs were incubated with hCAECs for 4 h. Results showed that both siRNA (orange signal in Figure 5R) and CD31 antibodies (red signal in Figure 5S) were successfully taken up by hCAECs and distributed in a non-homogeneous pattern in cytosol (Figures 5Q–T). The free siRNA-Cy3 not linked to MNPs was not obviously internalized into hCAECs (Figures 5A–D), suggesting the importance of the MNPs as nanocarriers to aid the cellular uptake of siRNA.

**FIGURE 6:**
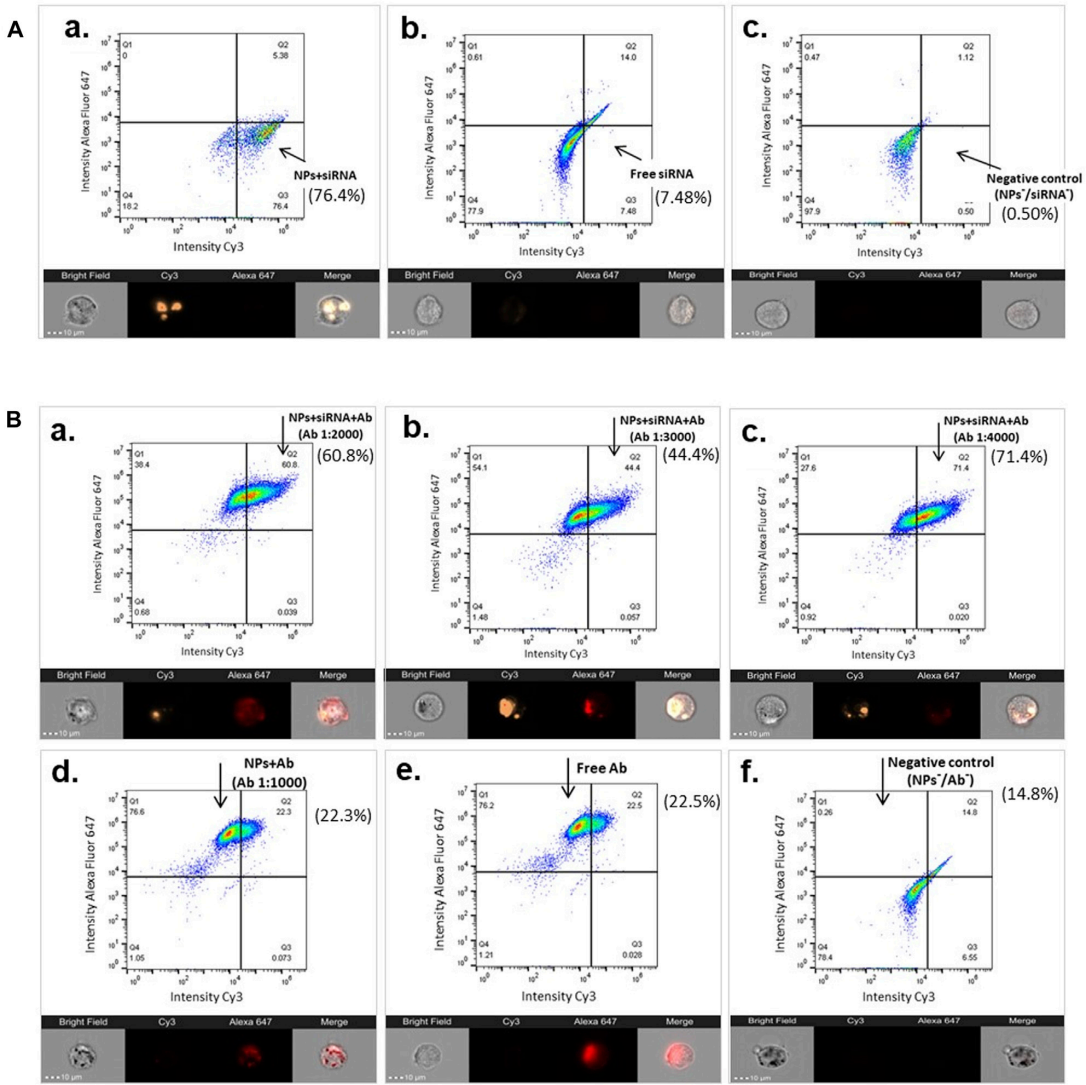
Image Stream flow cytometry showing cellular uptake of siRNA by functionalized MNPs. Magnetic Fe_3_O_4_ NPs were coated with trisodium citrate and further modified with PEI (MNP@Cit@PEI), and then complexed with GAPDH siRNA-Cy3 (siRNA), anti-CD31 antibody-AlexaFluor647 (Ab), or both, respectively. Functionalized MNPs were incubated with primary human coronary endothelial cells (hCAECs) for 2.5 h. Cellular uptakes of siRNAs were analyzed using Image Stream flow cytometry (top panels of each sub-figure) and images (bottom panels of each sub-figure). The percentage of siRNA-positive cells are indicated next to each of the flow cytometry panels. The gates were set using untreated cells. **(A)** Cellular uptake of siRNA-Cy3 MNPs (a), non-conjugated free siRNA-Cy3 (b), and untreated cells (negative control, c). **(B)** Cellular uptake of siRNA by Ab and siRNA dual-laden MNPs with different Ab dilutions 1:2000 (a), 1:3,000 (b), and 1:4,000 (c), as well as controls of MNP with Ab (d), free Ab (e), and untreated cells (f). On the Image Stream images, siRNA is visualized by Cy3 (orange), and CD31 antibody is visualized by AlexaFluor647 (red). Scale bar represents 10 µm.

The cellular uptake of the functionalized MNPs was also verified by ImageStream flow cytometry using samples from a typical preparation. The results supported the essential role of MNPs as nanocarriers for the cellular delivery of siRNA (Figure 6). About 76.4% of the hCAEC-engulfed siRNA was carried by MNP@Cit@PEI (Figure 6A-a) in comparison with ∼7.48% in the free siRNA control sample (Figure 6A-b). ImageStream images showed orange signal in the cytosol of hCAECs, indicating that siRNA-Cy3 carried by the MNPs were taken up by the cells (Figure 6A-a), whereas cells incubated with the free siRNA@Cy3 control had almost invisible Cy3 siRNA signal (Figure 6A-b). Using MNP@Cit@PEI@siRNA-Cy3 nanoparticles loaded with anti-CD31 antibody-AlexaFluor647 with three different Ab dilutions—1:2000, 1:3,000, and 1:4,000—a population of ∼60.8%, ∼44%, and ∼71%, respectively, of siRNA-positive cells was detected (Figure 6B a-c). Although the results were not entirely linear to the antibody dilutions, they do reveal a trend that more diluted antibodies favored the siRNA delivery.

### 3.5 Antibody conjugated MNPs achieved cell type specific delivery of siRNA under flow

**FIGURE 7:**
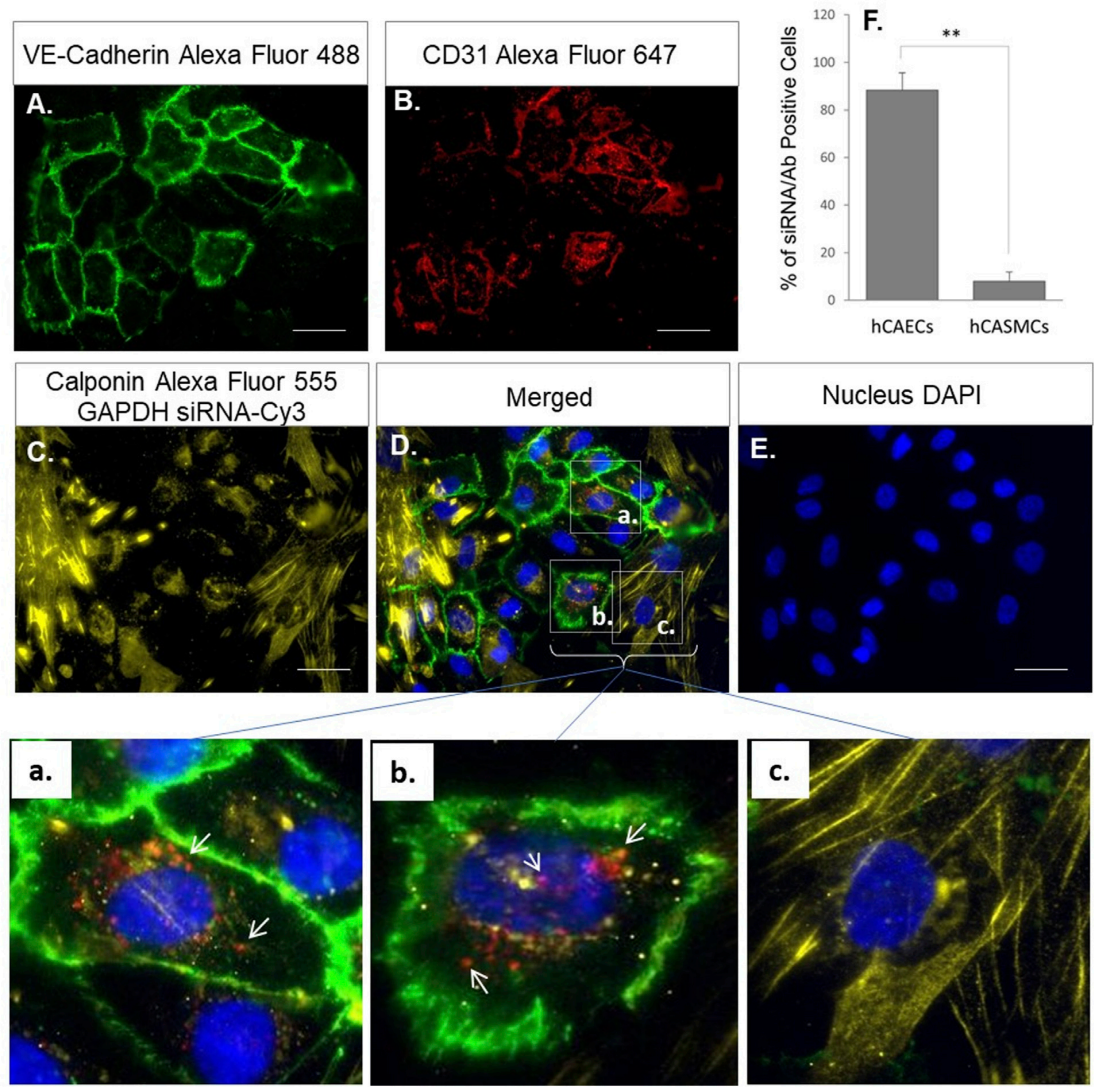
Endothelial-specific delivery of siRNA by CD31 antibody conjugated MNPs under flow. Primary human coronary artery endothelial cells (hCAECs) and vascular smooth muscle cells (hCASMCs) were co-cultured in ibidi µ-slides under laminar flow (10 dyn/cm^2^) to mimic blood circulation. Anti-CD31-Alexa Fluro 647 (red) and siRNA-Cy3 (yellow) conjugated magnetic Fe_3_O_4_ NPs (MNP@Cit@PEI) were then cultured with the cells for 24 h. Immunofluorescent staining of VE-Cadherin (green) **(A)** and Calponin (yellow) **(C)** and used to distinguish hCAECs and hCASMCs in the co-culture. Endothelial-targeted delivery of siRNA by CD31-antibody-conjugated MNPs was visualized under multi-channel fluorescent microscope. Cell nuclei were counterstained by DAPI **(E)**. a–c were digitally magnified corresponding areas in the merged image, showing that functionalized MNPs with both CD31 antibody (red) **(B)** and siRNA (yellow) **(C)** were specifically delivered into hCAECs (red or orange dots indicated by white arrows, a and b), but not in hCASMCs (c). Bar graph at top right **(F)** shows quantification of the percentage of hCAECs and hCASMCs that were siRNA/antibody positive.

To demonstrate the specificity of the CD-31-antibody-conjugated MNPs in targeting endothelial cells and their ability to be up taken by cells under blood flow, we employed the ibidi pump system where hCAECs and hCASMCs were co-cultured in a microchannel slide that were connected to the ibidi pump. The pump circulates cell culture medium as lamina flow through the co-cultured hCAECs and hCASMCs with a defined shear stress (10 dyn/cm^2^) to mimic the blood flow. The MNP@Cit@PEI@CD31-AF647@siRNA-Cy3 were added to the co-culture system for 24 h before fluorescent signals were visualized under a microscope (Figure 7). The results showed that hCAECs labeled by VE-cadherin (green, Figure 7A), CD31-Alexa Fluro 647 (red, Figure 7B), and hCASMCs labeled by Calponin (yellow, Figure 7C) were grown as a mixed population (Figure 7D) in the microchip. The zoomed-in images showed that the fluorescent signals CD31-Alexa Fluro 647 (red) and siRNA-Cy3 (yellow) carried by the MNP@Cit@PEI@CD31-AF647@siRNA-Cy3 were mostly present in the VE-cadherin-positive endothelial cells (Figures 7A, B) and not much in calponin-positive SMCs (Figures 7C, F); this suggested a selective targeting and delivery of siRNA into endothelial cells in a mixed cell population under flow. It is worth noting that calponin and miRNA-Cy3 were both yellow, but miRNA-Cy3 should always be together with the red CD-31 signal due to the co-conjugation to the MNPs. There was therefore no siRNA-Cy3 in SMCs since there were no red signals.

### 3.6 siRNAs carried by MNPs retain gene silencing function

**FIGURE 8:**
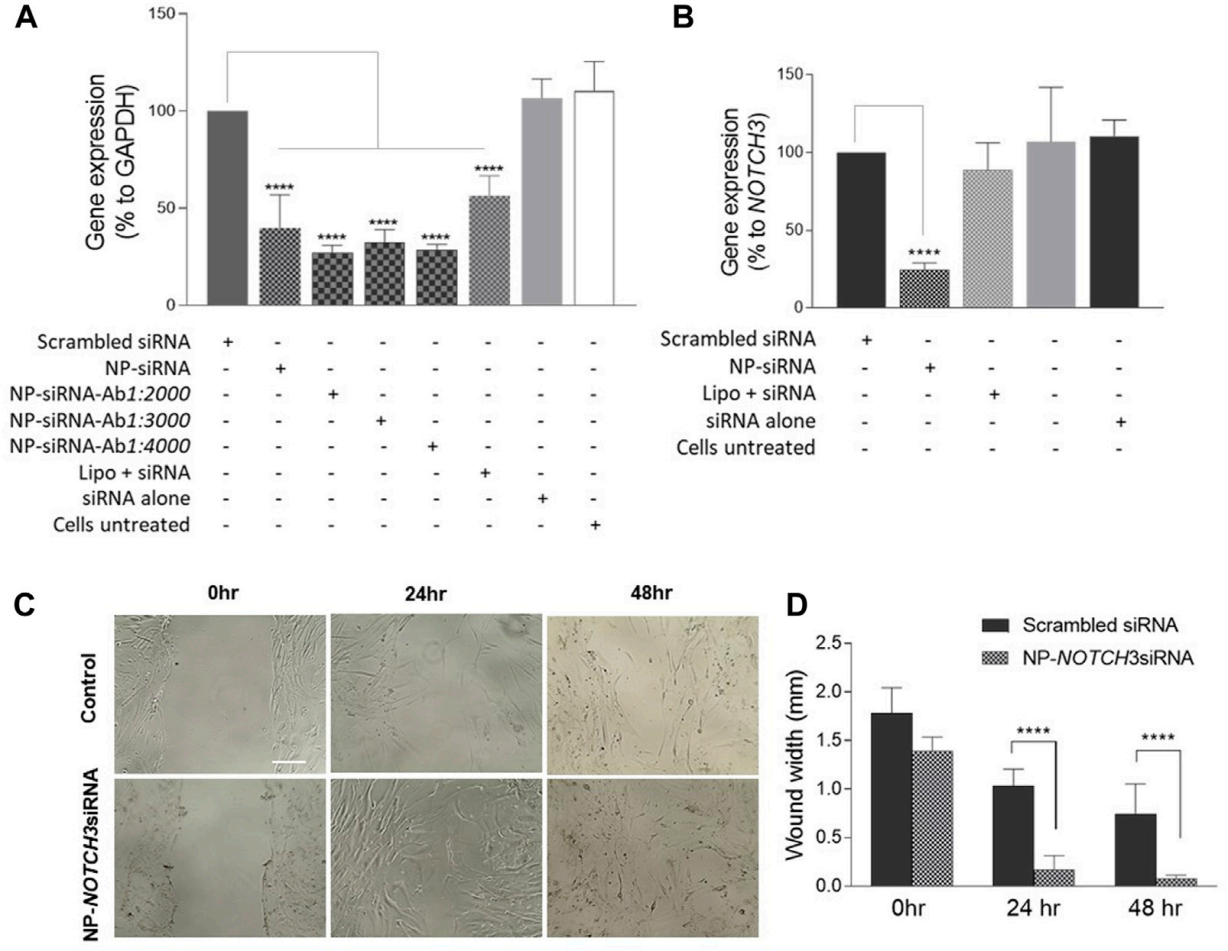
Functional characterization of siRNA conjugated magnetic Fe_3_O_4_ NPs by gene silencing. Human coronary artery endothelial cells (HCAECs) were incubated with MNP@Cit@PEI nanoparticles loaded with *GAPDH* siRNA and CD31 antibody, or free *GAPDH* siRNA for 48 h **(A)**. Human coronary artery smooth muscle cells (hCASMCs) were incubated with *NOTCH3* siRNA-loaded MNP@Cit@PEI nanoparticles for 48 h **(B)**. Cellular RNAs were extracted, and mRNA expression was determined by qRT-PCR. Free siRNAs were transfected using Lipofectamine 2000 (Lipo). The levels of GAPDH mRNA were normalized to 18S rRNA and presented as a percentage of *GAPADH* or *NOTCH3* expression relative to the untreated cells. Data are mean ± SE, n = 3. One-way ANOVA with *post hoc* tests, ****p < 0.0001. **(C)** Cell migration assay. HCASMCs were treated with *NOTCH3* siRNA-loaded MNP@Cit@PEI nanoparticles. Cell migration was determined by wound healing. **(D)** Quantification of cell migration data. Results shown as mean ± SE. Two-way ANOVA with Tukey’s *post hoc* test, ****p < 0.0001.

To verify the functional effectiveness of the MNP-loaded siRNA, hCAECs were incubated with the MNP@Cit@PEI@CD31-AF647@siRNA-Cy3 for 48 h, and the expression of *GAPADH* was analyzed by RT-qPCR. The results showed that the MNPs complexed with either *GAPDH* siRNA-Cy3 alone or with CD31 antibody could significantly reduce the expression of *GAPADH* in hCAECs (Figure 8A), suggesting a well-preserved function of the siRNA on the MNP carrier. To further verify the functionality of siRNAs on the MNP carrier, we loaded MNP@Cit@PEI with siRNA against the *NOTCH3* gene, which was delivered into hCASMCs. We chose the *NOTCH3* gene was because it is a well-known inhibitor of VSMC migration (Sweeney et al., 2004; Wang et al., 2008). The results showed that the *NOTCH3* siRNA MNPs significantly reduced *NOTCH3* expression in hCASMCs (Figure B). In line with our expectation, *NOTCH3* knockdown significantly reduced the migration of hCASMCs (Figures 8C, D).

### 3.7 Functionalized MNPs achieved site-specific delivery guided by magnet

**FIGURE 9:**
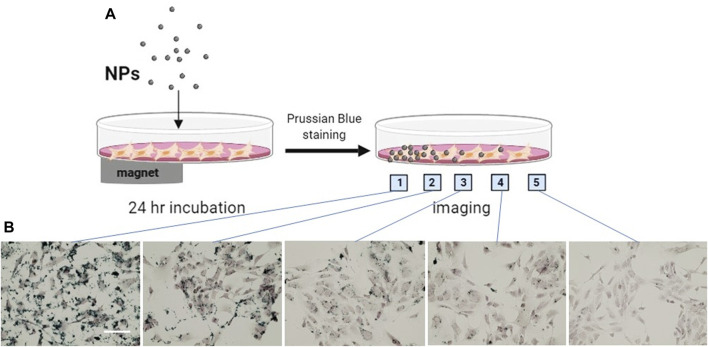
Magnetically guided delivery of magnetic Fe_3_O_4_ NPs (MNP@Cit@PEI). MNP@Cit@PEI nanoparticles were evenly applied to a monolayer human coronary artery endothelial cells (hCAECs) for 24 h with a magnet was placed beneath part of the cell culture plate as illustrated in the diagram **(A)**. Perls staining using Prussian blue was then carried out to detect magnetic guided iron distribution in hCAECs. Images were acquired at different positions of the cell culture dish as indicated **(B)**, using light microscope. Prussian Blue reagent stains iron (blue) and Nuclear Fast Red reagent stains nucleus (red). Scale bar represents 50 μm.

Taking advantage of the SPIO property of MNPs, we determined the site-specific delivery of the nanoparticles guided by a magnet. The MNP@Cit@PEI were applied to a monolayer of hCAECs, and a magnet was placed under the culture plate at one side (Figure 9A). Some 24 hours later, the MNPs were mainly distributed in or around cells where the magnet was placed, as shown by Prussian blue staining of iron (Figure 9B). This adds further to the ability of functionalized MPNs to target delivery of therapeutic agents, in addition to antibody guidance.

## 4 Discussion

This study generated Fe_3_O_4_ magnetic nanoparticles using chemical co-precipitation, and subsequently modified MNPs with trisodium citrate and PEI. We then developed a simple method to dually link antibodies and siRNA onto the citrate- and PEI-functionalized MNPs and achieved the targeted and efficient delivery of siRNA into human endothelial cells.

Citrate is a small molecule commonly used to stabilize colloidal metal nanoparticles, including MNPs (Arias et al., 2018; Franco-Ulloa et al., 2020). The multiple negatively-charged carboxylic acid groups on citrates provide repulsive forces to overcome Wan der Vaal forces in the nanoparticle solution to avoid aggregations (Lattuada and Hatton, 2007; Amina et al., 2024). The citrate ions are adsorbed on the nanoparticle surfaces by coordination via one or two carboxylates, leaving at least one carboxylic acid group exposed in the solution to interact with positively charged groups. Thus, sodium citrate cannot only be applied to enhance the stability of the nanoparticles but also link biomolecules like antibodies onto the surface of nanoparticles (Jazayeri et al., 2016).

It was hypothesized that when the pH of an antibody solution is lower than its isoelectric point (pI), the density of positive charges in the major plane of the antibody is usually high (Gupta et al., 2022). Under these conditions, the NH_2_ groups on lysine residues of the antibody are protonated, thereby generating NH3^+^. Thus, if the antibodies are in a solution with negatively charged nanoparticles, they most likely interact through their major plane with the surface of the nanoparticles. As the major plane of antibodies is away from the specific targeting (antigen-binding) region, such binding is likely orientated (Tam et al., 2017). This controlled approach ensures that the antigen binding sites are accessible. The isoelectric point of monoclonal IgG1 Ab ranges from 7.7 to 9.4 (Goyon et al., 2017; Yang et al., 2019). The anti-CD31 antibody employed in our research has a pI in this range, and the conjugation with biomolecules occurs in aqueous condition with pH 7.4. Thus, this approach can obtain oriented Ab conjugation (Figure 4A). Numerous studies have documented the antibody modification of MNPs for a wide range of biomedical applications, including imaging, drug delivery, and diagnosis (Pansieri et al., 2018). However, no report is apparent in the literature on citrate-modified iron oxide nanoparticles for antibody conjugation.

To effectively load negatively charged siRNA molecules onto MNPs, additional PEI functionalization of the citrate-coated MNP was carried out. PEI is a cationic polymer that is ideal for binding to the negatively charged siRNA molecules. The process of such complexation seems very efficient, and it has achieved nearly 100% uptake of free siRNAs in our reaction (Figure 4). It is worth noting that there was a sharp cut on the siRNA binding between 20 and 40 µg PEI-coated MNPs in the reaction. It is possible that we missed a transition phase between 20 and 40 µg provided, which is worth further investigation. Numerous studies have already used PEI to functionalize MNPs and then link siRNA for downstream applications, especially in tumor therapies (Wang et al., 2015; Wang et al., 2018). The conjugation occurs in an aqueous condition, without the need of expensive and complex cross-linkers that could be cytotoxic. An additional advantage of PEI is its “proton sponge” nature that could cause lysosome osmotic swelling and physical rupture, therefore protecting the siRNA from lysosomal degradation, which promotes endosomal escape (Boussif et al., 1995; Akinc et al., 2005; Casper et al., 2023). However, this study did not specifically investigate endosomal escape, which will be carried out in the future.

It is possible that the PEI layer electrostatically bound to the pre-coated citrate layer could potentially prevent the access of antibodies to the citrate molecules. However, surface coatings usually do not achieve 100% coverage. For example Atrei et al. (2022) analyzed the surface of MNP functionalized with citrate using XPS and ToF-SIMS and found that a single layer of citrate only covered a fraction of the nanoparticle’s surface, and the amount of citrate ions on the surface of the MNPs was independent of the fictionalization procedure. Therefore, citrate or PEI coatings are unlikely to entirely shield the nanoparticle surface, and the antibodies and siRNAs could make their way to form a complex with the materials. This was confirmed by the functional study on the cell model where the functionalized MNPs had successfully targeted human endothelial cells and delivered the siRNA with significant knockdown of the perspective genes (Figures 5–8).

Under static cell culture conditions, the functionalized MNPs were effectively taken up by either endothelial cells or VSMCs (Figures 5–8), likely through internalization or endocytic mechanisms which seemingly lack specificity. However, when the endothelial cells and VSMCs were co-cultured together under a physiological shear stress applied by a controlled flow, the CD31 antibody brought the MNPs specifically into endothelial cells (Figure 7). This suggests that the stringent flow condition that mimics *in vivo* settings selectively favors antibody-directed targeting and disadvantages the non-specific uptake of MNPs by other cell types. This finding is interesting and further stresses the importance of using correct model systems in human biomedical study.

This study used human endothelial cells and VSMCs as model cell types to evaluate the functionality and specificity of the functionalized MNPs. Endothelial cells are the inner layer of blood vessels. The pathology of endothelial cells could trigger inflammatory responses and contribute to a myriad of disease pathologies, ranging from cardiovascular and cerebral vascular diseases to cancer (Rajendran et al., 2013). The behavioral changes of VSMC—migration and proliferation—significantly contribute to the development of aging-related conditions such as atherosclerosis and coronary restenosis after angioplasty and stent implantation. Therapeutic tools in treating these conditions are limited and challenging. A targeted and controlled delivery of biomolecules using nanoparticles represent a promising platform (Pradhan-Nabzdyk et al., 2014), particularly biodegradable iron nanoparticles. CD31 antibody-conjugated nanoparticles that recognize vasculatures throughout the body could be used to develop therapeutic approaches that target systemic vascular conditions; however, for site-specific vascular pathologies, the advantage of the magnetic properties of MNPs could be used for local enrichment. For example, it has been challenging to deliver therapeutics into the brain due to the blood–brain barrier (BBB). Studies have demonstrated that an external magnetic field could promote MPNs to cross the BBB (Busquets et al., 2015; Chen et al., 2022). Additionally, the thermal energy released by MNPs under irradiation could increase BBB permeability and benefit drug delivery (Busquets et al., 2015). The antibody and siRNA dual loaded system could be extended to target other cell types and genes beyond the cardiovascular system.

### 4.1 Limitations

This study has limitations. For the cytotoxicity experiment, we observed significant reductions in the viability of VSMCs for all MNP concentrations after 24-h exposure. Although there was a trend of recovery after a prolonged 48-h cell culture, it would be ideal to conduct a long-term cytotoxicity analysis, ideally on animal models *in vivo*, which will done in future work. We did not study the details of the cellular trafficking of the MNPs and endosomal release of siRNA from the MNPs, which is important and will be of future interest. The current study is entirely performed *in vitro*, although human primary cells were employed. We do not know whether the particles will be stable in the blood *in vivo*, with a good “stealth effect” (Wen et al., 2023). We also do not know the stability of the siRNA itself when exposed *in vivo*. The rapid clearance of nanomaterials by the reticuloendothelial system has been a concern in nanomedicine (Li and Huang, 2009; Wen et al., 2023). Should this be the case for our functionalized MNPs, PEGylation of the siRNA or the nanocomposite would be a promising way forward to improve the stability and retention time of nanoparticles in the blood (Jung et al., 2010; Wen et al., 2023), but the effect of PEGylation on the exposure of antibodies and subsequent antigen recognition would still need to be carefully evaluated. PEGylation could also reduce the potential cytotoxicity of PEI polymer (Casper et al., 2023), improve the stability of the nanocomposite (Sung et al., 2003), and enhance endosomal escape (Li and Kataoka, 2021). Finally, apart from demonstrating the functional effectiveness of the antibody- and siRNA-loaded MNPs in gene knockdown in human vascular cells, the material of the fully functionalized MNPs were not characterized in our study. Future work will thoroughly analyze the physiochemical properties of these nanoparticles.

## 5 Conclusion

This work demonstrated an effective complexation of anti-CD31 antibody and siRNA onto magnetic MNP@Cit@PEI nanoparticles and achieved a targeted delivery of siRNA into human endothelial cells and the functional silencing of specific target genes. This novel and simplified antibody and siRNA dual-loading method opens possibilities of attaching siRNA and different antibodies onto MNP surfaces for biomedical applications without the need for expensive and complex cross-linkers.

## Data Availability

The original contributions presented in the study are included in the article/supplementary material; further inquiries can be directed to the corresponding author.
